# A comparative study of prenatal development in *Miniopterus schreibersii fuliginosus, Hipposideros armiger *and *H. pratti*

**DOI:** 10.1186/1471-213X-10-10

**Published:** 2010-01-21

**Authors:** Zhe Wang, Naijian Han, Paul A Racey, Binghua Ru, Guimei He

**Affiliations:** 1 School of Life Sciences, East China Normal University, Shanghai 200062, PR China; 2 Institute of Zoology, Chinese Academy of Sciences, Beijing 100101, PR China; 3 School of Biological Sciences, University of Aberdeen, Aberdeen AB24 2TZ, UK

## Abstract

**Background:**

Bats comprise the second largest order of mammals. However, there are far fewer morphological studies of post-implantation embryonic development than early embryonic development in bats.

**Results:**

We studied three species of bats (*Miniopterus schreibersii fuliginosus, Hipposideros armiger *and *H. pratti*), representing the two suborders Yangochiroptera and Yinpterochiroptera. Using an established embryonic staging system, we identified the embryonic stages for *M. schreibersii fuliginosus, H. armiger *and *H. pratti *and described the morphological changes in each species, including the development of the complex and distinctive nose-leaves in *H. armiger *and *H. pratti*. Finally, we compared embryonic and fetal morphology of the three species in the present study with five other species for which information is available.

**Conclusion:**

As a whole, the organogenetic sequence of bat embryos is uniform and the embryos appear homoplastic before Stage 16. Morphological differentiation between species occurs mainly after embryonic Stage 16. Our study provides three new bat species for interspecific comparison of post-implantation embryonic development within the order Chiroptera and detailed data on the development of nose-leaves for bats in the superfamily Rhinolophoidea.

## Background

**Figure 1:**
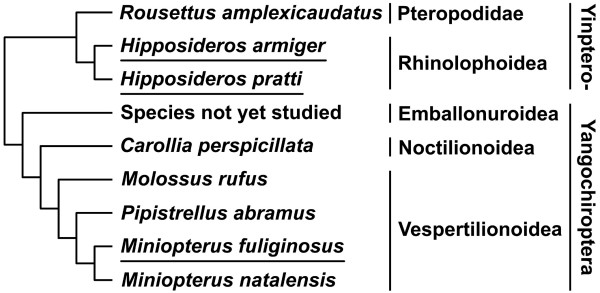
**Phylogeny of the eight species of bats studied for embryogenesis**. Species studied in this paper are underlined. Yinptero-, Yinpterochiroptera.

The Chiroptera is the second largest order of mammals with over 1100 species [1]. It consists of two suborders and five superfamilies [2]. Although early development has been described for many bat species [3], post-implantation staging systems have been described for only five species from three superfamilies (Fig. 1) [4-8]. However, an important superfamily of bats, the Rhinolophoidea, in the suborder Yinpterochiroptera, has not been studied. Bats in this superfamily are characterised by the complex structure of their nose-leaves which are associated with echolocation [9,10]. In this paper, we applied the staging system developed by Cretekos et al [5] to three bat species, *M. schreibersii fuliginosus, H. armiger *and *H pratti*, which belong to the superfamilies Vespertilionoidea and Rhinolophoidea respectively [2], to investigate whether there are any species specific differences in embryonic development.

*M. schreibersii fuliginosus*, Schreibers's long-fingered bat, is widespread from the temperate zone to the tropics in the Old World [11,12]. In the north temperate zone (32.5°N and 33.5°N), copulation and fertilization occur in autumn, and implantation and embryonic development are delayed for the duration of hibernation [13,14]. Normal embryonic development proceeds after arousal from hibernation in spring and parturition occurs in summer [15].

*H. armiger*, the great leaf-nosed bat, is widely distributed in subtropical and tropical zones of Asia [12]. At lower latitudes, this species hibernates from December to February [16,17] and embryonic development is delayed [18].

*H. pratti*, Pratt's leaf-nosed bat, has been found in a few Asian countries and is distributed across the same latitudes as *H. armiger *[12]. Although this species was described in 1891 [19], it has been the subject of few studies and none on reproduction or development.

Here we compare morphological changes during embryonic and fetal development for the three bat species, describing the differentiation and development of the face and limbs, and comparing them with information already available for five other bat species. By adding the two hipposiderid species studied in this paper, representatives of another superfamily have been considered (Fig. 1).

## Results

### Specimen summary

Female *M. schreibersii fuliginosus*, *H. armiger *and *H. pratti *possess bicornuate uteri and are monotocous. *H. armiger *were in torpor on 19 Feb 2009 and 4 March 2008. On other capture dates, the three species of bats had already aroused. We obtained embryos from 24 *M. schreibersii fuliginosus *in nine stages of development (Stages 13-18, 20, 23 and the fetal stage), 26 *H. armiger *in 11 stages (Stages 10, 11, 14, 17-23 and fetal stage) and 12 *H. pratti *in nine stages (Stages 11, 14-16, 19, 20, 22, 23 and the fetal stage) and three neonates of *M. schreibersii fuliginosus*. Because they were resected for mRNA extraction, the limbs of a few specimens are missing (Stages 20, 21 of *H. armiger *and Stages 19, 20, 23 of *H. pratti*) and some limb information is lacking. Table 1 shows the capture dates of the female bats and the date on which each stage was dissected for each species. All the prenatal stages in this study occurred between April and June. In all gravid *M. schreibersii fuliginosus *examined, a fetus was present in the right horn of uterus, whereas in all gravid *H. armiger *and *H. pratti *it was in the left horn of uterus. The crown rump length (CRL) and uterus diameter (UD) progressively increased during embryonic development (Table 2).

**Table 1:** Capture and dissection dates of the *M. schreibersii fuliginosus *, *H.armiger *and *H. pretti*.

	*M. schreibersii fuliginosus*		*H. armiger*					*H. pratti*		
			
Stage	04/24/09	04/28/08	02/19/09	03/04/08	04/24/09	04/28/08	05/22/08	04/24/09	05/22/08	06/04/09
10					04/25/09 (2)					
11					04/25/09 (2)			04/25/09 (1) 05/05/09 (1)		
12										
13		04/30/08 (1) 05/02/08 (1)								
14		05/02/08 (1) 05/04/08 (1) 05/06/08 (1)		04/25/09 (1)				05/02/09 (1)		
15		05/04/08 (2) 05/05/08 (1)						05/11/09 (1)		
16		05/03/08 (1) 05/06/08 (2) 05/07/08 (1)						05/05/09 (1)		
17		05/03/08 (1) 05/07/08 (2)			05/05/09 (1)					
18		05/06/08 (1) 05/14/08 (1)	04/02/09 (2)	04/14/08 (1)						
19				04/18/08 (1)			05/22/08 (1)		05/22/08 (2)	
20		05/18/08 (1) 05/20/08 (1)		04/21/08 (1) 04/22/08 (1)					05/22/08 (2)	
21						05/14/08 (2)				
22				04/22/08 (1) 04/26/08 (1)		05/14/08 (1)		05/22/09 (1)		
23		05/21/08 (1)		04/26/08 (1) 04/29/08 (1)	05/14/08 (1)				05/22/08 (1)	
Fetal	05/28/09 (1) 06/23/09 (2)	05/22/08 (1)	04/25/09 (1)	05/13/08 (1) 05/14/08 (1)	05/11/09 (1)		05/22/08 (1)		06/04/09 (1)	

Dates are given as month/day/year. Dates in the second line of the table are the capture dates, and other dates are the dissection dates. The number of embryos or fetuses is shown in the brackets.

**Table 2:** Feature comparisons of eight bat species during embryonic and fetal development.

		*C. perspicillata*^a^			*H. armiger*^b^			*H. pratti*^b^			*R. amplexicaudatus*^c^	
					
Stage	Commen features	Specific features	UD	CRL	Specific features	UD	CRL	Specific features	UD	CRL	Specific features	CRL
10	Neural tube and somite formation	4-12 somite pairs			5-12 somite pairs	7.00 ± 1.10 (2)						
11	Rostral neuropore closure; cranial flexure; 2 pharyngeal arches; optic evagination; otic vesicle	13-20 somite pairs			22 somite pairs; cervical flexure	7.42 ± 0.14 (2)	2.42 ± 0.03 (2)	22 somite pairs; cervical flexure	7.17 ± 0.02 (2)	2.69 ± 0.35 (2)		
12	Forelimb buds form; tail bud forms; caudal neuropore closes; 3 pharyngeal arches	21--29 somite pairs; cervical flexure	5.75 ± 0.64	3.4 ± 0.42								
13	Oral groove; optic cup; lens placode; hindlimb bud; forelimb AER	30-35 somite pairs										
14	Retinal pigment; genital tubercle	36-40 somite pairs; small nasal pits; propatagium and plagiopatagium primordia; hindlimb AER	6.95 ± 0.44	5.35 ± 0.24	36 somite pairs; round nasal pits	9.58 (1)	5.14 (1)	40 somite pairs; round nasal pits	8.86 (1)	5.52 (1)		
15	Lens vesicles; auditory hillocks	Hand and foot plates; external auditory meatus	8.65 ± 1.20	7.45 ± 0.92				40 somite pairs; hand plate	11.10 (1)	7.80 (1)		
16	Naris; pinna	Eyelid primordium; nose-leaf primordium; tragus; vibrissal folicles; forelimb digital condensations; uropatagium primordium	12.06 ± 1.45	8.66 ± 1.05				Nose-leaf primordium; external auditory meatus; pinna; antitragus; foot plate	12.90 (1)	9.52 (1)	Vibrissal folicles; antitragus; calcar primordium; forelimb digital condensations; hindlimb interdigit tissue receding	8.5 (2)
17		Eyes begin to close; tongue out; chin wart; unicorn bump; hindlimb interdigit tissue receding	13.45 ± 1.34	9.15 ± 1.34	Hindlimb interdigit tissue receding; uropatagium primordium	15.00 (1)	12.38 (1)				Hindlimb interdigit tissue receding	9.4 (1)
18	Head and body smoother, rounder	Free thumb; free toes; calcar; flexure at wrist; uropatagium enclose the whole tail	16.32 ± 0.98	12.35 ± 1.16	12 pairs of ribs; eyes begin to close; 1st supplementary leaflet; frontal sac primordium; free thumb; free toes; calcar	17.05 ± 0.43 (3)	13.11 ± 0.44 (3)				Oral papillae; free thumb; free toes; claw primordia	13.4 (1)
19		Claw primordia			2nd supplementary leaflet; flexure at wrist; claw primordia; uropatagium enclose the whole tail	18.98 ± 1.02 (2)	15.66 ± 0.81 (2)	1^st ^supplementary leaflet; vibrissal folicles; uropatagium enclose the whole tail		16.79 ± 1.68 (2)		16.1 (1)
20	Distal forelimbs overlap over face	Eyelids closed	20.0 ± 3.54	16.35 ± 1.06	Eyelids closed; 3rd supplementary leaflet; vibrissal folicles	20.13 ± 0.71 (2)	18.50 ± 0.32 (2)	Eyelids closed; nose-leaves achieve adult appearance		18.95 ± 0.07 (2)	Eyelids closed	16.3-20.7 (5)
21						22.52 ± 0.34 (2)	19.39 ± 0.25 (2)					
22	Wing membranes corrugated	Nose-leaf; eyelids reopening; claws pigmented, hooked	23.03 ± 2.68	20.02 ± 0.26	Claws pigmented, hooked	23.78 ± 1.68 (3)	22.04 ± 0.37 (3)	Claws pigmented, hooked	23.04 (1)	21.44 (1)	Genitals begin dimorphic differentiation	17.2-28.6 (3)
23						25.77 ± 0.57 (3)	23.58 ± 0.13 (3)			24.86 (1)		
Fetal	Vibrissae; short hair widespread on dorsal parts	Eyes completely open	23.53 ± 0.64	21.13 ± 0.06	Nose-leaves achieve adult appearance; genitals begin dimorphic differentiation	38.58 ± 6.82 (5)	36.48 ± 5.36 (5)	Genitals begin dimorphic differentiation	34.23 (1)	33.31 (1)	Pigmentation in palpebral line	27.3-44.4 (5)

		***M. schreibersii fuliginosus*^b^**			***M. natalensis*^d^**			***P. abramus*^e^**		***M. rufus*^f^**		
					
**Stage**	**Commen features**	**Specific features**	**UD**	**CRL**	**Specific features**	**UD**	**CRL**	**Specific features**	**CRL**	**Specific features**	**UD**	**CRL**

10	Neural tube and somite formation							7 somite pairs	2.3			
11	Rostral neuropore closure; cranial flexure; 2 pharyngeal arches; optic evagination; otic vesicle							10-12 somite pairs; cervical flexure	2.3-2.6			
12	Forelimb buds form; tail bud forms; caudal neuropore closes; 3 pharyngeal arches				27 somite pairs	5.8 ± 0.12 (3)	3.7 ± 0.94 (3)	16-18 somite pairs	2.1-2.2			
13	Oral groove; optic cup; lens placode; hindlimb bud; forelimb AER	31 somite pairs	6.31 ± 0.38 (2)	4.22 ± 0.11 (2)		8.0 ± 2.09 (11)	5.2 ± 0.26 (11)	25-28 somite pairs; hindlimb AER; long nasal pits	2.6-4.1	33-36 somite pairs	5.0 (1)	5.0 (1)
14	Retinal pigment; genital tubercle	42 somite pairs; long nasal pits; propatagium and plagiopatagium primordia; hindlimb AER	8.39 ± 0.48 (3)	5.85 ± 0.49 (3)	Long nasal pits; propatagium and plagiopatagium primordia; hindlimb AER	9.0 ± 1.01 (18)	5.9 ± 0.80 (18)	Hand plate	4.1-5.7	37-40 somite pairs; long nasal pits; propatagium primordia; hindlimb AER	8.0-9.5 (3)	5.8-7.0 (3)
15	Lens vesicles; auditory hillocks	42 somite pairs; 10 pairs of ribs; external auditory meatus; forelimb digital condensations; foot plate	9.92 ± 1.47 (3)	7.19 ± 0.71 (3)	Forelimb digital condensations; foot plate	10.4 ± 0.81 (21)	7.3 ± 0.75 (21)	Plagiopatagium primordium; foot plate	5.9	Plagiopatagium primordia; hand and foot plates	10-10.5 (2)	7.2-9.0 (2)
16	Naris; pinna	Eyelid primordium; tragus; uropatagium primordium	11.27 ± 0.52 (4)	8.28 ± 0.48 (4)	Tragus; hindlimb digital condensations; hindlimb interdigit tissue receding; uropatagium primordium	11.8 ± 0.83 (26)	9.1 ± 0.68 (26)	Tragus; hindlimb interdigit tissue receding	5.6-7.2	Antitragus; vibrissal folicles; uropatagium primordium; fore- and hindlimb digital condensations	10.0-12.0 (2)	9.7-10.0 (2)
17		Eyes begin to close; vibrissal folicles; chin wart; hindlimb interdigit tissue receding	12.96 ± 0.78 (3)	9.59 ± 0.54 (3)	Vibrissal folicles	12.2 ± 1.02 (31)	10.5 ± 0.63 (31)	Vibrissal folicles; free thumb; free toes; propatagium	9.1	cervical flexure absent; fore- and hindlimb interdigit tissue receding	11.0 (1)	11.4 (1)
18	Head and body smoother, rounder	Free thumb; free toes; flexure at wrist; calcar	13.88 ± 1.95 (2)	10.48 ± 1.56 (2)	Free thumb	13.0 ± 1.33 (10)	12.0 ± 0.80 (10)	Eyelids closed; claw primordia	10.6-11.2	Eyes begin to close; free thumb; claw primordia	12.0-13.0 (4)	12.0-13.5 (4)
19		Claw primordia			Claw primordia; calcar	13.1 ± 1.28 (4)	12.8 ± 0.69 (4)					
20	Distal forelimbs overlap over face	Eyelids closed; tooth primordium; uropatagium enclose the whole tail	17.46 ± 1.12 (2)	14.44 ± 0.82 (2)	Eyelids closed; uropatagium enclose the whole tail	14.3 ± 0.35 (2)	14.4 ± 0.82 (2)	Claws hooked; uropatagium enclose the whole tail	12.7	Eyelids closed	13.5-14.5 (2)	16.7-18.5 (2)
21					Claws pigmented, hooked	15.3 (1)	15.7 (1)				19.0 (1)	19.0 (1)
22	Wing membranes corrugated									Eyelids reopening; teeth formed	19.0 (1)	28.0 (1)
23		Genitals begin dimorphic differentiation	18.90 (1)	17.30 (1)								
Fetal	Vibrissae; short hair widespread on dorsal parts		22.99 ± 4.71 (4)	23.09 ± 5.91 (4)					17.4			

UD: uterus diameter (mm); CRL: crown rump length (mm); AER: apical ectodermal ridge. The average UD and CRL are given with standard deviation or the range of measurements. The number of specimens is given in the parenthesis.

^a ^Data from Cretekos *et al*. [5]; ^b ^Data from current study; ^c ^Data from Giannini *et al*. [7]; ^d ^Data from Hockman *et al*. [4]; ^e ^Data from Tokita [6]; ^f ^Data from Nolte *et al*. [8].

### Embryonic and fetal development of *M. schreibersii *fuliginosus

**Figure 2:**
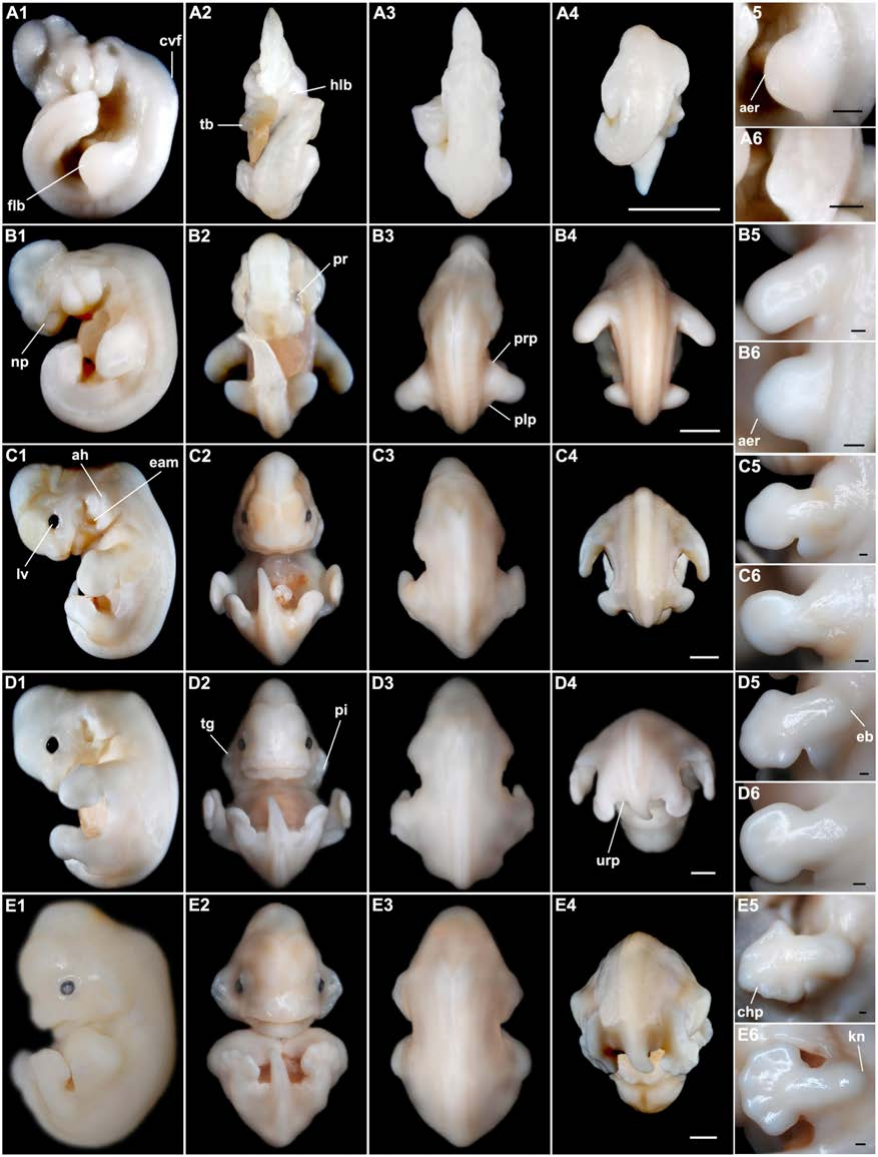
***M. schreibersii fuliginosus *at embryonic Stages 13-17**. (A1-6) Stage 13. (B1-6) Stage 14. (C1-6) Stage 15. (D1-6) Stage 16. (E1-6) Stage 17. (A1, B1, C1, D1, E1) Lateral view with dorsal to the right; (A2, B2, C2, D2, E2) Ventral view; (A3, B3, C3, D3, E3) Dorsal view of the head and trunk; (A4, B4, C4, D4, E4) Dorsal view of the trunk and tail; (A5, B5, C5, D5, E5) Close-up for the left forelimb; (A6, B6, C6, D6, E6) Close-up for the left hindlimb. aer, apical ectodermal ridge; ah, auditory hillocks; cvf, cervical flexure; chp, chiropatagium; eam, external auditory meatus; eb, elbow; flb, forelimb bud; hlb, hindlimb bud; kn, knee; lv, lens vesicle; np, nasal pit; og, oral groove; plp, plagiopatagium; pi, pinna; pr, pigmented retina; prp, propatagium; tb, tail bud; tg, tragus; urp, uropatagium. Bar = 1 mm in A4, B4, C4, D4 and E4 (applies to A1-4, B1-4, C1-4, D1-4, E1-4); bar = 200 μm in A5-6, B5-6, C5-6, D5-6 and E5-6.

**Figure 3:**
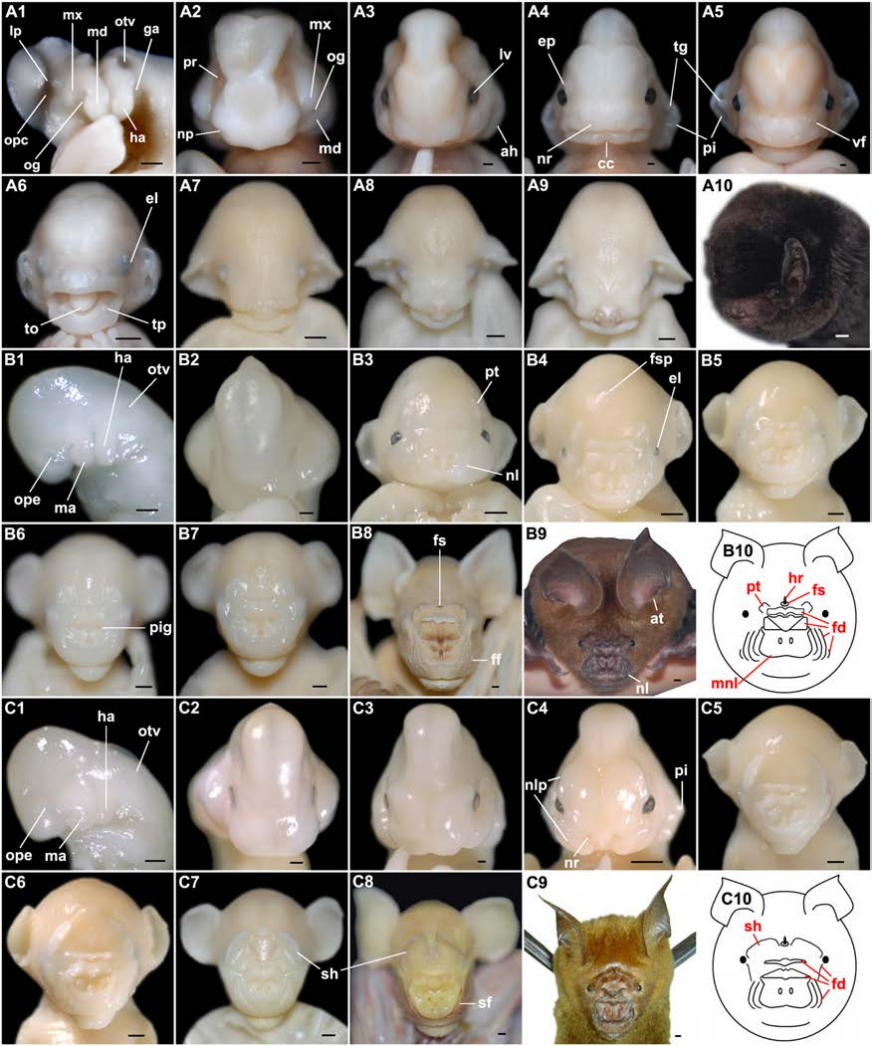
**Craniofacial development of *M. schreibersii fuliginosus*, *H. armiger *and *H. pratti***. (A1-10) M. schreibersii fuliginosus: (A1) Lateral view of left side of Stage 13 head; (A2-10) Face-on views of Stage 14 (A2), Stage 15 (A3), Stage 16 (A4), Stage 17 (A5), Stage 18 (A6), Stage 20 (A7), Stage 23 (A8), fetal stage (A9), and adult (A10) heads. (B1-10) *H. armiger*: (B1) Lateral view of left side of Stage 11 head; (A2-9) Face-on views of Stage 14 (B2), Stage 17 (B3), Stage 18 (B4), Stage 19 (B5), Stage 20 (B6), Stage 22 (B7), fetal stage (B8), and adult (B9) heads; (B10) A diagram of an adult *H. armiger*'s face illustrating nose-leaves. (C1-10) *H. pratti*: (C1) Lateral view of left side of Stage 11 head; (C2-9) Face-on views of Stage 14 (C2), Stage 15 (C3), Stage 16 (C4), Stage 19 (C5), Stage 20 (C6), Stage 22 (C7), fetal stage (C8), and adult (C9) heads; (C10) A diagram of an adult *H. pratti*'s face illustrating nose-leaves. ah, auditory hillocks; at, antitragus; cc, chin cleft; el, eyelid; ep, eyelid primordium; fd, fold; ff, the 4th fold; fs, frontal sac; fsp, frontal sac primordium; ga, glossopharyngeal arch; ha, hyoid arch; hr, hair; lp, lens placode; lv, lens vesicle; md, mandible; mx, maxilla; ma, mandibular arch; mnl, main nose-leaf; nl, nose-leaf; nlp, nose-leaf primordium; np, nasal pit; nr, naris; og, oral groove; ope, optic evagination; opc, optic cup; otv, otic vesicle; pi, pinna; pig, pigment; pr, pigmented retina; pt, protuberance; sf, the second fold; sh, shield; tg, tragus; to, tongue; tp, tooth primordium; vf, vibrissal follicles. Bar = 200 μm in A1-5, B1-2 and C1-3; bar = 1 mm in A6-9, B3-8 and C4-8; bar = 2 mm in A10, B9 and C9.

Stage 13 (Fig. 2A; Fig. 3A1): 31 pairs of somites were evident early in this stage. The body was curved significantly so that the head touched the tail bud. The cervical flexure was obvious at the junction of the head and trunk. A cleft (oral groove) divided the first pharyngeal arch into the maxilla and the mandible. The second (hyoid) arch was as large as the mandible, whereas the third (glossopharyngeal) arch almost disappeared behind the hyoid. The optic cups, lens placodes, and otic vesicle were clearly visible at the lateral side of the head. The dilated forelimb bud presented an apical ectodermal ridge (AER) at its distal edge. A pair of hindlimb buds began to appear near the tail bud.

Stage 14 (Fig. 2B; Fig. 3A2): specimens at Stages 14-15 had a maximum of 42 pairs of somites, so that somitogenesis was completed with 42 pairs before Stage 15. The cervical flexure inscribed a right angle. Pigmentation in the retina began and the genital tubercle appeared below the umbilicus. A pair of nasal pits was evident, as a long groove. The primordium of the propatagium and plagiopatagium, which will form part of the wing membrane, appeared at the two sides of the forelimb bud. The shape of the hindlimb bud was very similar to that of the forelimb bud in Stage 13 and its AER emerged.

**Figure 4:**
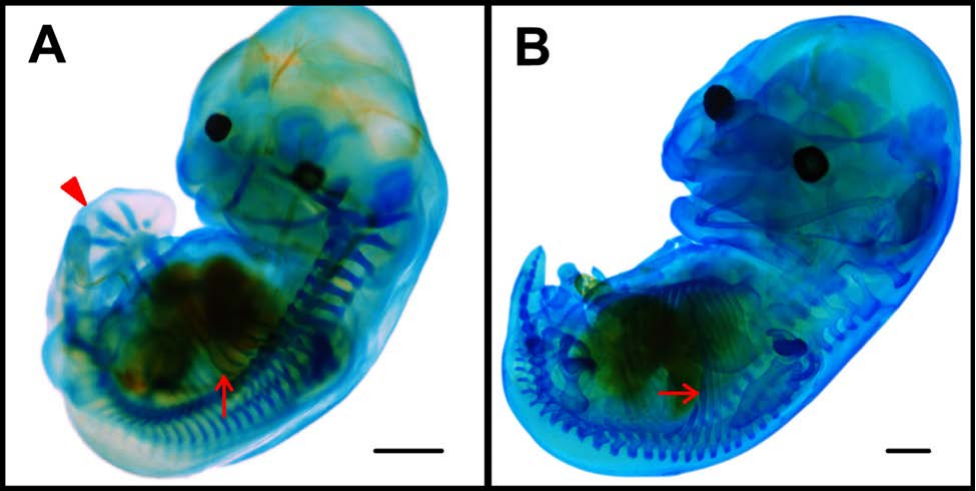
**Alcian blue staining**. (A) *M. schreibersii fuliginosus *at Stage 15. (B) *H. armiger *at Stage 18 (limbs removed). Red arrows indicate the ribs and the triangle indicates the left hand plate with digit condensation. Bar = 1 mm.

Stage 15 (Fig. 2C; Fig. 3A3): ribs were visualised by Alcian blue and ten pairs of ribs were evident (Fig. 4A). The body became straighter. The eyes were black and lens vesicles were evident. The external auditory meatus and auditory hillocks replaced a small part of the first pharyngeal arch and the second pharyngeal arch. The lower jaw was clearly visible at the lateral side. Discoid hand plates and foot plates were formed. Digit condensations, which will become the skeleton of the digits, began in the hand plate (Fig. 4A). The primordium of the propatagium and plagiopatagium continued to extend.

Stage 16 (Fig. 2D; Fig. 3A4): eyelid primordia and a pair of nares were formed. A muzzle consisting of the nares, upper jaw and the lower jaw was easily recognized. There was an evident cleft in the center of the lower jaw. The auditory hillocks became pinnae and tragi, the former curling inward at the distal tips. Elbows could be recognized as flexures on the forelimbs. The interdigital notches were distinctly present in the hand plate. The primordium of the uropatagium emerged between the legs and the tail.

Stage 17 (Fig. 2E; Fig. 3A5): vibrissal follicles appeared at the two sides of the nose and chin warts were seen on the lower jaw. The eyelids began to cover the eyes. The muzzle extended. The curled pinnae spread out and the tragi enlarged. Knees could be recognized as a flexure on the hindlimb. Tissues between all the digits of the hindlimb and between the first and second digits of the forelimb began to regress, whereas those between other digits of the forelimb remained to form the chiropatagium.

**Figure 5:**
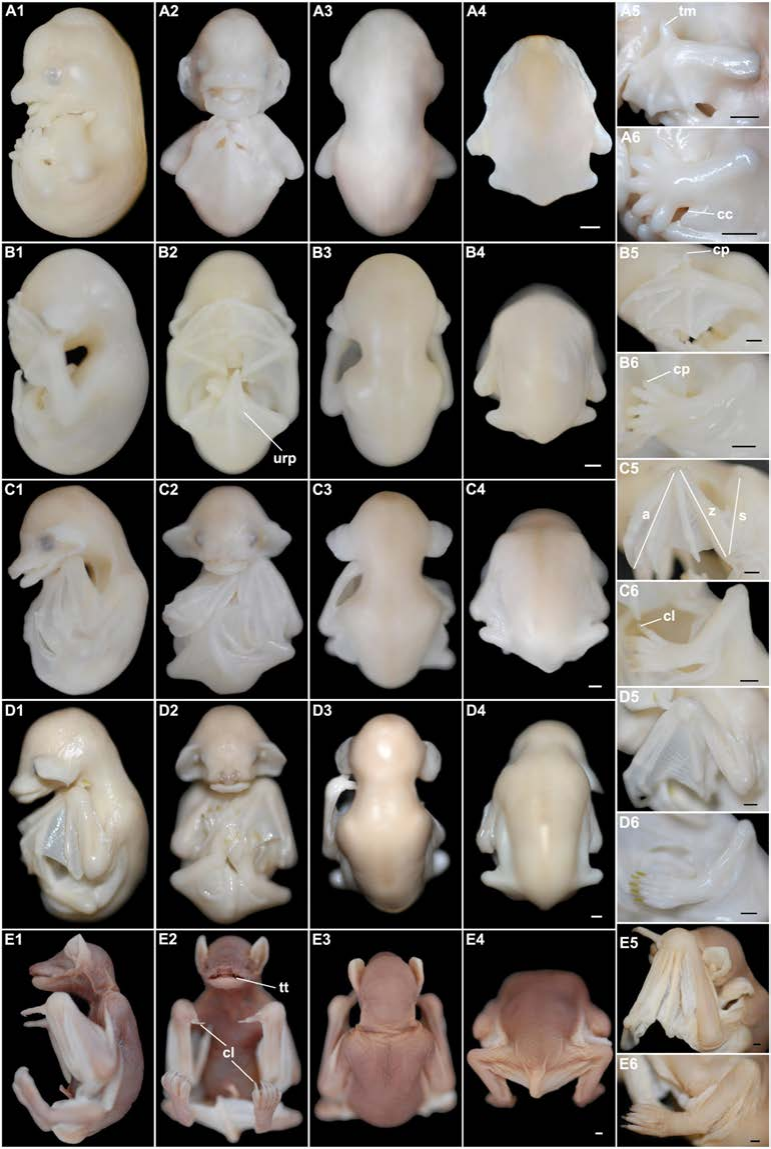
***M. schreibersii fuliginosus *at embryonic Stages 18-23, the fetal stage and a newborn *M. schreibersii fuliginosus***. (A1-A6) Stage 18. (B1-B6) Stage 20. (C1-C6) Stage 23. (D1-D6) fetal stage. (E1-E6) newborn. (A1, B1, C1, D1, E1) Lateral view with dorsal to the right; (A2, B2, C2, D2, E2) Ventral view; (A3, B3, C3, D3, E3) Dorsal view of the head and trunk; (A4, B4, C4, D4, E4) Dorsal view of the trunk and tail; (A5, B5, C5, D5, E5) Close-up for the left forelimb; (A6, B6, C6, D6, E6) Close-up for the left hindlimb. a, autopod; cc, calcar; cl, claw; cp, claw primordium; s, stylopod; tm, thumb; tt, tooth; urp, uropatagium; z, zeugopod. Bar = 1 mm in A4, B4, C4, D4, E4 (applies to A1-4, B1-4, C1-4, D1-4, E1-4), A5-6, B5-6, C5-6, D5-6 and E5-6.

Stage 18 (Fig. 5A; Fig. 3A6): the whole body appeared rounder, because the cervical flexure disappeared and the back of the head became round. Pigmentation appeared on the face and the eyelids almost covered the eyes with only a small opening. A pair of tooth primordia was evident on the lower jaw. The first digit of the forelimb (hallux) was almost free and other digits of the forelimb began to elongate within the chiropatagium. Flexure at the wrist was easily recognized. Tissues between the digits of the foot disappeared and the calcar appeared at the ankle.

Stage 20 (Fig. 5B; Fig. 3A7): the eyes were completely closed at the beginning of this stage. The main features of the craniofacial morphology had achieved an adult appearance (Fig. 3A10). Claw primordia were not seen at Stage 18, but were enlarged at Stage 20, suggesting their presence at Stage 19. The pinna grew large and straight. The hands with chiropatagia were enlarged, nearly covering the entire face and overlapping at the anterior edges. The feet overlapped each other. The uropatagium enclosed the whole tail and extended to the ankle.

Stage 23 (Fig. 5C; 3A8): 2 hair follicles were visible on each upper eyelid. The genital tubercle had become a vagina or a penis so gender could be distinguished. The chiropatagium, plagiopatagium and uropatagium became slack and thin as they increased in area. In the forelimb, the autopod was a little longer than the stylopod and zeugopod (a = 8.95 mm; z = 8.21 mm; s = 8.03 mm).

Fetal stage (Fig. 5D; Fig. 3A9): pigmentation around the nares and on the claw became fuscous and the pigmentation of the entire body surface increased as fetal development progressed.

Neonates (Fig. 5E): 3 *M. schreibersii fuliginosus *were born in the captive colony on July 5, 7 and 17, with eyelids closed. Their mean body weight was 3.22 ± 0.12 g and their mean forearm length was 16.26 ± 0.05 mm. The skin was faintly red in colour. Short and dense whiskers and vibrissae were present around the mouth and on the cheeks. Two long hairs projected from each upper eyelid. Exiguous hairs were also found on the legs, elbows, digits of the hindlimbs and the first digit of the forelimb. Very small teeth were present and the tongue was obvious. Claws on the digits of the hindlimbs and the first digit of the forelimb had been keratinized and were sufficiently aculeate to allow the neonate to attach to the mother.

### Embryonic and fetal development of *H. armiger*

**Figure 6:**
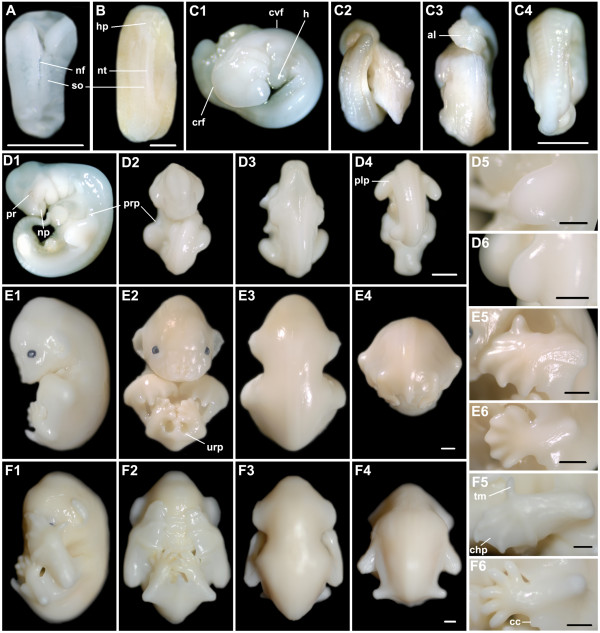
***H. armiger *at embryonic Stages 10-18**. (A) Dorsal view of a five-somite embryo at Stage 10. (B) Dorsal view of a twelve-somite embryo at Stage 10. (C1-4) Stage 11. (D1-6) Stage 14. (E1-6) Stage 17. (F1-6) Stage 18. (C1, D1, E1, F1) Lateral view with dorsal to the right; (C2, D2, E2, F2) Ventral view; (C3, D3, E3, F3) Dorsal view of the head and trunk; (C4, D4, E4, F4) Dorsal view of the trunk and tail; (D5, E5, F5) Close-up for the left forelimb; (D6, E6, F6) Close-up for the left hindlimb. al, allantois; cc, calcar; chp, chiropatagium; crf, cranial flexure; cvf, cervical flexure; h, heart; hp, head process; nf, neural fold; np, nasal pit; nt, neural tube; plp, plagiopatagium; pr, pigmented retina; prp, propatagium; so, somite; tm, thumb; urp, uropatagium. Bar = 1 mm in B, C4, D4, E4, F4 (applies to C1-4, D1-4, E1-4, F1-4), E5-6 and F5-6; bar = 500 μm in A and D5-6.

Stage 10 (Fig. 6A, B): Two specimens with five and 12 somites were obtained at this stage. The neural fold was open at the anterior region in the five-somite embryo. It then fused to form the neural tube. At the end of this stage, the neural tube was clearly seen from the dorsal view of the twelve-somite embryo.

Stage 11 (Fig. 6C; Fig. 3B1): had 22 pairs of somites. The embryo curved strongly to make a round body shape. The caudal region with a spherical allantois flexed to the right side of the head. The rostral neuropore was closed and the cranial and cervical flexures became apparent. Optic invaginations which would become optic cups were at the lateral side of the forebrain. The first two pharyngeal arches (mandibular and hyoid) and otic vesicles were formed at the lateral sides of the hindbrain.

Stage 14 (Fig. 6D; Fig. 3B2): had 36 pairs of somites. The cervical flexure inscribed a right angle. Pigmentation in the retina began and the genital tubercle below the umbilicus appeared. A pair of nasal pits was evident and appeared as a round depression. The length was equal to the width in the forelimb bud but shorter than the width in the hindlimb bud. The primordium of the propatagium and plagiopatagium appeared at the two sides of the forelimb bud. The hindlimb AER emerged.

Stage 17 (Fig. 6E; Fig. 3B3): a small part of the lower jaw could be seen from the front view of the face although much of it was hidden by the upper jaw. A pair of protuberances was present close to the eyes. In the middle of the face, the leaf-like folds of skin (nose-leaves) were shaped around the nares, although they were still rudimentary. Tissues between all the digits of the hindlimb and between the first and second digits of the forelimb regressed halfway, whereas those between other digits of the forelimb remained to form the chiropatagium. The primordium of the uropatagium emerged between the legs and the tail.

Stage 18 (Fig. 6F; Fig. 3B4): the eyelids had begun to cover the eyes. The nose-leaves became evident. There was a transverse fold and the main trough-like nose-leaf had nostrils in the middle. One fold (or supplementary leaflet) was seen on each side of the cheek. The primordium of the frontal sac was present in the middle of the forehead. The thumb was totally free and other digits on the hand had elongated. Free toes and the calcar were present. Alcian blue staining revealed 12 pairs of ribs at this stage (Fig. 4B).

**Figure 7:**
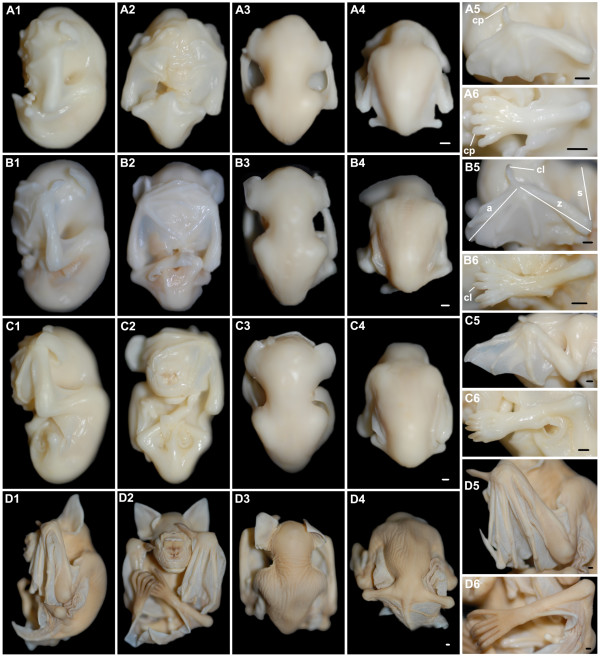
***H. armiger *at embryonic Stages 19-23 and the fetal stage**. (A1-A6) Stage 19. (B1-B6) Stage 22. (C1-C6) Stage 23. (D1-D6) fetal stage. (A1, B1, C1, D1) Lateral view with dorsal to the right; (A2, B2, C2, D2) Ventral view; (A3, B3, C3, D3) Dorsal view of the head and trunk; (A4, B4, C4, D4) Dorsal view of the trunk and tail; (A5, B5, C5, D5) Close-up for the left forelimb; (A6, B6, C6, D6) Close-up for the left hindlimb. a, autopod; cl, claw; cp, claw primordium; s, stylopod; z, zeugopod. Bar = 1 mm in A4, B4, C4, D4 (applies to A1-4, B1-4, C1-4, D1-4), A5-6, B5-6, C5-6 and D5-6.

Stage 19 (Fig. 7A; Fig. 3B5): more than half the face was obscured by the hand plate. The eyelids half covered the eyes at the beginning of this stage and covered most of the eyes at the end. A second fold was seen on each side of the cheek. Flexure at the wrist was apparent. Knob-like claw primordia were evident at the tip of each toe and the thumb. The legs overlapped each other. The uropatagium enclosed the whole tail by the end of this stage.

Stage 20 (Fig. 3B6): eyelids covered the eyes completely. Pigmentation was evident around the nostrils. The third fold was seen on each side of the cheek. Vibrissal follicles emerged between the main nose-leaf and the mouth.

Stage 21: the overall appearance of the embryo was similar to the former stages. The nose-leaves, protuberances and the frontal sac were larger and more evident than before.

Stage 22 (Fig. 7B; Fig. 3B7): the chiropatagium, plagiopatagium and uropatagium became slack and thin as they grew. In the forelimb, the autopod was shorter than the stylopod and zeugopod (a = 9.88 mm; z = 10.25 mm; s = 10.15 mm).

Stage 23 (Fig. 7C): the overall appearance of the embryo was similar to the previous stages. In the forelimb, the autopod was a little longer than the stylopod and zeugopod (a = 13.35 mm; z = 12.34 mm; s = 12.30 mm). The entire forehead and half the face was covered by one hand with large chiropatagia between the digits.

Fetal stage (Fig. 7D; Fig. 3B8): the genital tubercle had become a vagina or a penis so gender could be distinguished. In early fetal development, fuscous pigments appeared around the nostrils. The fourth fold on each side of the cheek could be clearly seen so that the nose-leaves had achieved an adult appearance (Fig. 3B9-10). A hollow occurred in the middle of the frontal sac and it appeared earlier in the male fetuses than in the females. Pigmentation of the entire body surface increased as fetal development progressed. During late fetal development, short hairs emerged from the hollow of the frontal sac; short whiskers and vibrissae were present around the mouth and on the nose-leaves; exiguous hairs were found on the legs, arms, the dorsal surface of the tail, uropatagium, toes and thumb. Claws were sharp and keratinized.

### Embryonic and fetal development of *H. pratti*

**Figure 8:**
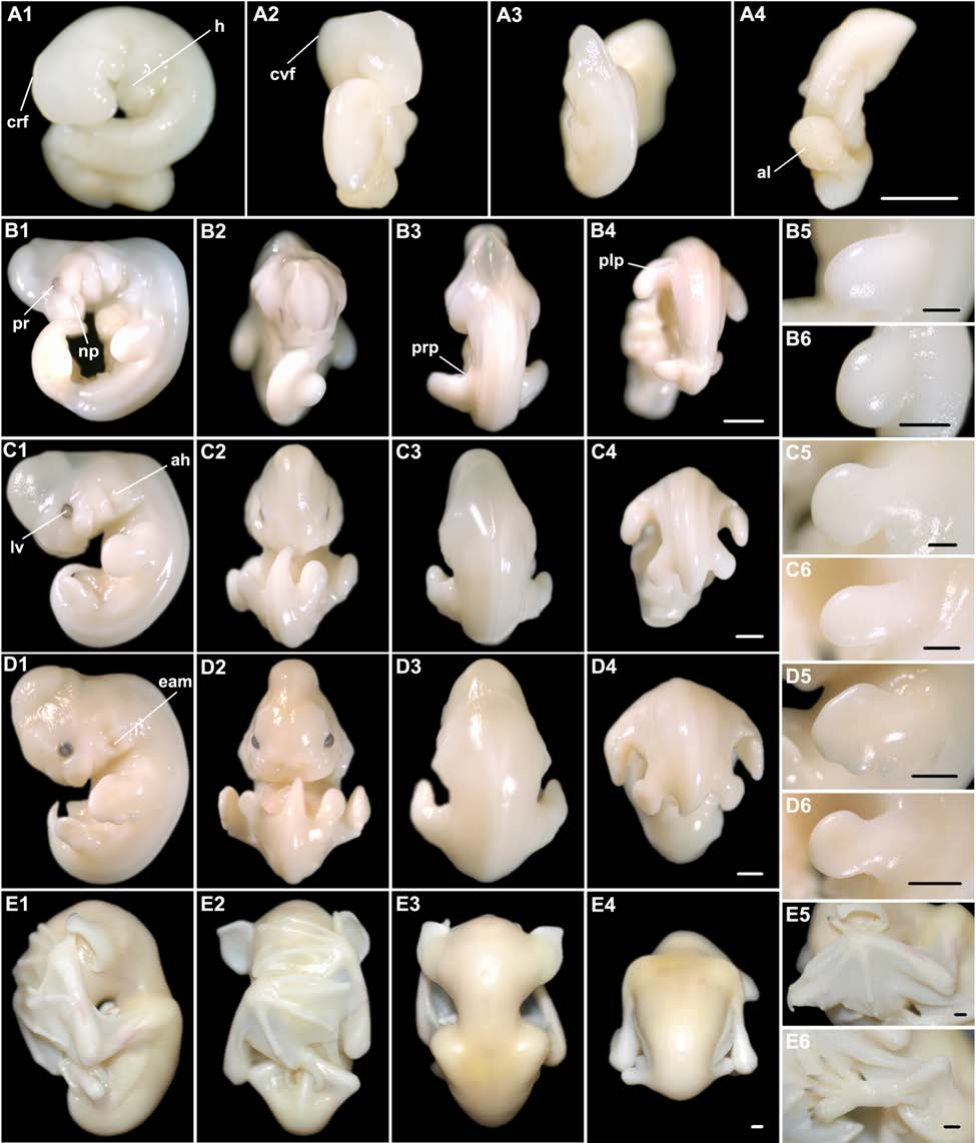
***H. pratti *at embryonic Stages 11-22**. (A1-4) Stage 11. (B1-6) Stage 14. (C1-6) Stage 15. (D1-6) Stage 16. (E1-6) Stage 22. (A1, B1, C1, D1, E1) Lateral view with dorsal to the right; (A2, B2, C2, D2, E2) Ventral view; (A3, B3, C3, D3, E3) Dorsal view of the head and trunk; (A4, B4, C4, D4, E4) Dorsal view of the trunk and tail; (B5, C5, D5, E5) Close-up for the left forelimb; (B6, C6, D6, E6) Close-up for the left hindlimb. ah, auditory hillocks; al, allantois; crf, cranial flexure; cvf, cervical flexure; eam, external auditory meatus; h, heart; lv, lens vesicle; np, nasal pit; plp, plagiopatagium; pr, pigmented retina; prp, propatagium. Bar = 1 mm in A4, B4, C4, D4, E4 (applies to A1-4, B1-4, C1-4, D1-4, E1-4), D5-6 and E5-6; bar = 500 μm in B5-6 and C5-6.

Stage 11 (Fig. 8A; Fig. 3C1): the characters of the embryo were very similar to *H. armiger *at Stage 11 (see above results).

Stage 14 (Fig. 8B; Fig. 3C2): specimens at Stages 14-15 had a maximum of 40 pairs of somites, so that somitogenesis was completed with 40 pairs before Stage 15. The length was longer than the width in the forelimb bud but shorter than the width in the hindlimb bud. Other characters were the same with *H. armiger *at Stage 14 (see above results).

Stage 15 (Fig. 8C; Fig. 3C3): the body became straighter. Pigmentation in the retina became darker and lens vesicles were evident. A wide depression, which became the external auditory meatus, was apparent between the first pharyngeal arch and the second, and above which the auditory hillocks were formed. Discoid hand plates were clearly seen, but foot plates were not evident. The primordium of the propatagium and plagiopatagium continued to extend.

Stage 16 (Fig. 8D; Fig. 3C4): a pair of nares was distinct. Nose-leaf primordia emerged beside the nares and above the eyes. The upper jaw and the lower jaw were formed, but the lower jaw was so short that it was hidden beneath the upper jaw. The external auditory meatus appeared and the auditory hillocks became pinnae and antitragus. The interdigital notches were distinctly evident in the hand plate. Foot plates were also evident and similar to the hand plates at Stage 15.

Stage 19 (Fig. 3C5): the eyelids half covered the eyes. A transverse bilobed shield between the eyes was enlarged. The nose-leaves were evident. As with *H. armiger*, *H. pratti *had a transverse fold and a main nose-leaf in the middle of the face. Beside the main nose-leaf, one fold was seen on each side of the cheek. The primordium of the frontal sac was present in the middle of the forehead. Vibrissal follicles emerged between the main nose-leaf and the mouth. The uropatagium enclosed the whole tail by the end of this stage.

Stage 20 (Fig. 3C6): eyelids covered the eyes completely. The second fold and a fold under the eyes had formed on each side of the cheek so that the nose-leaves had achieved an adult appearance (Fig. 3C9-10).

Stage 22 (Fig. 8E; Fig. 3C7): in the forelimb, the autopod was shorter than the stylopod and zeugopod (a = 10.08 mm; z = 11.80 mm; s = 11.17 mm).

Stage 23: the overall appearance of the embryo was similar to the former stages. The shield between the eyes was larger but the genital tubercle was maintained.

Fetal stage (Fig. 3C8): the genital tubercle had become a vagina or a penis so gender could be distinguished. A hollow occurred in the middle of the frontal sac. Clear differences were evident in noseleaf morphology between *H. pratti *and *H. armiger *at this stage (Fig. 3B8). Pigmentation of the entire body surface increased as fetal development progressed. Claws were sharp and keratinized.

## Discussion

For the three species in this study, late embryonic stages and fetal stages were obtained mainly from April to June (Table 1). Embryonic stages of *H. armiger *captured on 19 Feb 2009 and 4 March 2008 were more advanced than those captured on 24 April 2009, 28 April 2008 and 22 May 2008. That is, nearly all the embryos from the bats captured during torpor (or hibernation) developed faster than those captured after arousal. We captured dozens of *H. armiger *from the same cave and maintained them for another study. When kept cool during January and February, they became torpid even if food was provided. This suggests that these bats hibernated as did populations of the same species at lower latitudes (23°N) [16,17]. Although accelerated development could be the result of individual variation in the population, it is most likely due to the fact that the bats captured on 19 Feb 2009 and 4 March 2008 were artificially aroused from hibernation, kept in an elevated ambient temperature and supplied with food. Similar phenomena also occurred in other bat species, including *M. schreibersii fuliginosus *[20,21].

As a whole, the organogenetic sequence of bat embryos is uniform and the embryos appear homoplastic before Stage 16 when the nose-leaf begins to form in some species. There are many common features at the same stage for all the bat species studied, summarized in table 2. The column "common features" contains the key features that can be used to identify the different stages of bat development. When these common features occur, the corresponding stage starts or is in progress. For example, neural tube and somite formation represent the beginning of Stage 10. It is difficult to find common features for all eight bat species at Stages 17, 19 and 23, because some studies did not identify stages strictly according to the staging system developed by Cretekos et al [5] and some features occurred earlier or later in *C. perspicillata *than in other species. In some instances, for example Stage 15, the common features are listed as lens vesicles and auditory hillocks. Although these terms were not present in the descriptions of all the bat species considered, we listed them for most species in which these features are described.

However, there are also many specific features for each species. Besides the CRL, UD and somite count, the most profound developmental differences are expected to be in craniofacial development, which is more complex in species possessed of nose-leaves (*H. armiger*, *H. pratti *and *C. perspicillata*). The earliest differences are evident early in embryonic Stage 14 when nasal pits are identified. In *H. armiger *and *H. pratti *these appear as round depressions, whereas in *C. perspicillata *they are relatively small and in other species are long grooves. More evident differences of the face start from Stage 16 when the nose-leaf primordia are formed in *H. armiger*, *H. pratti *and *C. perspicillata*. Although *H. armiger *and *H. pratti *are phylogenetically close, their nose-leaves develop distinct morphologies. There are four main differences of nose-leaves between these two species. First of all, *H. armiger *develops four folds on each side of the cheek, whereas *H. pratti *develops two. Secondly, *H. pratti *develops an evident fold under each eye, whereas *H. armiger *does not. Thirdly, the transverse bilobed shield between the eyes is much larger in *H. pratti *than in *H. armiger*. Finally, the part of the main fold above nostrils is bigger in *H. armiger *than in *H. pratti*. In contrast, *M. schreibersii fuliginosus *and *M. natalensis *are also phylogenetically close and their embryonic development and adult morphology are similar.

The eyelids of all eight bat species close before Stage 20, but two of them (*C. perspicillata *and *M. rufus*) reopen at embryonic Stage 22 and the others never open during prenatal development. The uropatagium does not enclose the whole tail in *M. rufus *and *R. amplexicaudatus*, but does so in other species. Many other differences occur mainly in the timing of organogenesis. For example, the foot plate formed one stage later in *H. pratti *than in other species. The uropatagium encloses the whole tail later in *M. schreibersii fuliginosus*, *M. natalensis *and *P. abramus *than in *C. perspicillata *because of the longer tails in the former three species.

## Conclusion

Morphological differentiation of bat species is completed prenatally and occurs mainly after embryonic Stage 16 when a muzzle consisting of the nares, nose-leaves (if present), upper jaw and the lower jaw is formed. Our study provides three new bat species for interspecific comparison of post-implantation embryonic development within the order Chiroptera and detailed data on the development of nose-leaves for bats in the superfamily Rhinolophoidea.

## Methods

### Phylogeny construction

An illustration of phylogenic relationships of the eight bat species, which were studied for embryonic staging systems, was represented based on previous publications [2,22].

### Animal collection and breeding

All procedures involving animals were carried out in accordance with the Policy on the Care and Use of Animals, approved by the Ethical Committee, State Key Laboratory of Reproductive Biology, Institute of Zoology, Chinese Academy of Sciences. Several ten thousands of *M. schreibersii fuliginosus*, *H. armiger *and *H.pratti *roost in a large cave at Anhui province of China (30°20.263'N, 117°50.180'E). During an investigation of infectious diseases of bats, a total of 75 female *M. schreibersii fuliginosus*, *H. armiger *and *H.pratti *were captured using hand or mist nets on 4 March 2008, 28 April 2008, 22 May 2008, 19 Feb 2009, 24 April 2009 and 4 June 2009.

After capture, these bats were kept in a flight room (4 × 4 × 2.5 m) which was covered with wire netting on the wall and roof to allow the bats to hang. The room was kept dark all day and the temperature was maintained between 18 and 24°C. Plastic bowls (12 × 7 × 5 cm) for water and food hung on the wall and were 12 cm from the roof. Water was freely available. A diet of mealworms mixed with powdered multivitamin and calcium tablets was provided in the bowls from 2000 h to 0800 h. In the beginning, some of the bats needed to be fed by hand but after several days, they could find food by themselves, like the others.

### Specimen processing

Bats in the captive colony were euthanized by decapitation. The reproductive tract was then dissected, measured, and further dissected to expose the conceptus. After dissection, specimens were fixed overnight in Bouin's fluid then washed with several changes of 70% ethyl alcohol and stored at room temperature until use. Before being photographed, specimens were cleared in 0.1% ammonium hydroxide in 70% ethyl alcohol. The whole bodies of the specimens were photographed from the lateral view, ventral view and dorsal view. Close-up of the forelimb, hindlimb and face were also photographed.

### Staging bat embryos

Because the bats were already pregnant when they were caught, it was difficult to determine the exact duration of pregnancy. Thus, bat embryos were staged using the system developed by Cretekos et al [5] according to morphological characteristics. In addition, the uterus diameter (UD, the maximal diameter of each gravid uterine horn) was measured during dissection. Crown rump lengths (CRL) and stylopod, zeugopod and autopod lengths were measured after fixation and ribs were counted after skeletal analysis.

### Skeletal analysis

Embryos of *M. schreibersii fuliginosus *at Stage 15 and *H. armiger *at Stage 18 were fixed overnight in Bouin's solution and cartilage stained with Alcian blue. The detailed staining method for the bat embryo is the same as for the mouse embryo [23].

### Comparison of features of eight bat species

We summarized features in each stage for the three bat species from this study and used the data of other five species from previous publications [4-8]. If all or most of the species possess the same feature and this feature is not described in other stages, it was classified as "common features", and others were classified as "specific features".

## Authors' contributions

ZW conceived and designed the study, identified the stages of bat embryos, and drafted the manuscript. NH carried out the experiments and fed the bats. PAR participated in the preparation of figures and critically revised the manuscript. BR participated in the preparation of figures. GH helped to draft the manuscript. All authors read and approved the final manuscript.
